# A Hybrid Auricular Framework of Autologous Rib Cartilage and a Porous Polyethylene Implant for Reconstruction of Congenital Microtia: A Modification of Nagata's Technique

**DOI:** 10.1089/fpsam.2022.0152

**Published:** 2024-01-08

**Authors:** Peter K.M. Ku, Alexander C. Vlantis, Marcus C. Tong, Trevor T.T. Chan, Zenon W.C. Yeung, Ryan H.W. Cho, Thomas S.C. Hui, Osan Y.M. Ho, Iris O.S. Leung, Willis S.S. Tsang, Nelson K.L. Lai, Wai-tsz Chang, Victor Abdullah, Andrew van Hasselt, Michael C.F. Tong

**Affiliations:** ^1^ Department of Otorhinolaryngology—Head and Neck Surgery, Division of Facial Plastic Surgery, United Christian Hospital and Tseung Kwan O Hospital, Hong Kong SAR, China.; ^2^ Department of Otorhinolaryngology, Head and Neck Surgery, Prince of Wales Hospital, The Chinese University of Hong Kong, Hong Kong SAR, China.; ^3^ Department of Otorhinolaryngology—Head and Neck Surgery, United Christian Hospital and Tseung Kwan O Hospital, Hong Kong SAR, China.

## Abstract

**Background:**

An implant (porous polyethylene) is an alternative to rib cartilage for microtia reconstruction but carries a risk of extrusion.

**Objective:**

To evaluate the outcome of a hybrid framework of implant with rib cartilage for microtia reconstruction.

**Methods:**

Patients who underwent Nagata's technique for microtia reconstruction were reviewed for complications and aesthetic score. In stage 1, a rib cartilage framework or a hybrid framework of implant with rib cartilage was used. In stage 2, the framework was elevated and supported by an implant for projection. Postoperative outcomes were reported for both groups.

**Results:**

Forty-four ears of 40 patients underwent surgery. Eleven ears received a rib auricular framework and 33 ears a hybrid auricular framework. The mean postoperative follow-up for the rib and hybrid groups was 76.3 and 43.1 months, respectively. No supporting postauricular implant extruded, whereas stainless-steel wires extruded in seven ears (15.9%). Five (15.2%) hybrid frameworks were removed due to infection or extrusion. Mean operating time was 2 h shorter in the hybrid group. Aesthetic outcomes were similar for both groups.

**Conclusion:**

A hybrid framework of rib and implant that requires less harvested cartilage is feasible for microtia reconstruction, but caution should be used due to its higher explantation rate.

**KEY POINTS**

**Question:** Can an implant be used to augment rib cartilage to reconstruct a congenitally deformed auricle?

**Findings:** When a hybrid of rib with implant was used to reconstruct microtias, less operating time and less harvested rib cartilage were required than with rib alone, aesthetic outcomes were universally good, but the explantation rate was higher in the hybrid group.

**Meaning:** This proof of concept demonstrated that microtia reconstruction can be achieved using less rib cartilage by combining it with an implant, which may allow surgery to be done in younger patients and in those needing bilateral reconstructions that can be done simultaneously. However, caution should be used when choosing a hybrid versus a rib-only approach.

## Introduction

Implants, of porous polyethylene for instance, have been used in microtia reconstruction since the 1980s with encouraging results.^1–3^ Implants overcome the limitations of the supply of autologous rib cartilage (rib), allowing microtia reconstruction to be performed as single-stage surgery in patients younger than 8 years, as both less rib cartilage and only one surgery are needed.^4–7^ The criticism of implants is their reported 3.7–30% risk of extrusion, which is undesirable in microtia reconstruction despite the use of vascularized temporoparietal fascia to protect them.^8–10^

Wu et al. reported a series of nine single-stage microtia reconstructions using rib with implants to construct a composite or hybrid auricular framework.^11^ They formed the ninth rib cartilage into a helix and sutured it to an auricular implant prosthesis (Stryker, Kalamazoo, MI, USA) and reported acceptable cosmetic results with no extrusions during a 2-year follow-up. Subsequently, Wang et al.,^12^ Kim et al.,^13^ and Shan et al.^14^ used an implant (Stryker) as the postaural wedge to support the rib auricular framework, and covered the postaural defect with vascularized temporoparietal fascia using the classical Nagata's reconstructive technique.

Their modification spared harvesting additional rib during the second stage of reconstruction and achieved acceptable auricular projection without subsequent implant extrusion. These reports suggest that an implant is feasible when incorporated with rib that reduces the risk of extrusion. In our study, we hypothesize that there is no difference in the aesthetic outcome of Nagata's microtia reconstruction using rib versus a hybrid, with both having comparable complications, but with the hybrid requiring less rib cartilage.

## Materials and Methods

### Subject recruitment for the study

We conducted a retrospective study at the United Christian Hospital (UCH) and Tseung Kwan O Hospital (TKOH), tertiary referral centers for otorhinolaryngology—head and neck surgery and academic units of the Chinese University of Hong Kong. The study was approved by the institutional review board of the Kowloon East Hospital Cluster (KC/KE-21-0147/ER-3), which oversees research activities in these institutions. Patients with congenital microtia who received primary or revision microtia reconstructive surgery between January 2012 and December 2019 in either UCH or TKOH were included. Patients who defaulted follow-up after surgery or who had incomplete photographic documentation of their auricular condition for assessment were excluded.

We reviewed hospital charts for demographic data, surgical technique, operating time, intraoperative, and postoperative complications. We deemed a pneumothorax, skin necrosis, infection, hypertrophic scar formation, auricular framework exposure or fracture, extrusion of stainless-steel wires, and extrusion or explantation of the framework to be complications.

### Surgical techniques for external ear reconstruction

#### Nagata's technique for microtia reconstruction

The classical Nagata's stage 1 consists of rib harvest, auricular incisions, construction of the rib auricular framework using the sixth to ninth rib cartilages, placement of the auricular framework under an auricular skin flap, and transposition of the earlobe.^15–17^ Stage I was modified by adding an implant to the rib to create a hybrid auricular framework.

The classical Nagata's stage 2 carves a wedge of rib, harvested during the second stage, to support and provide auricular framework projection, covered with vascularized temporoparietal fascia and an overlying skin graft. Nagata's stage 2 was modified by using a wedge of implant instead of more harvested rib cartilage avoiding a second chest wound.^18^

**Fig. 1.**
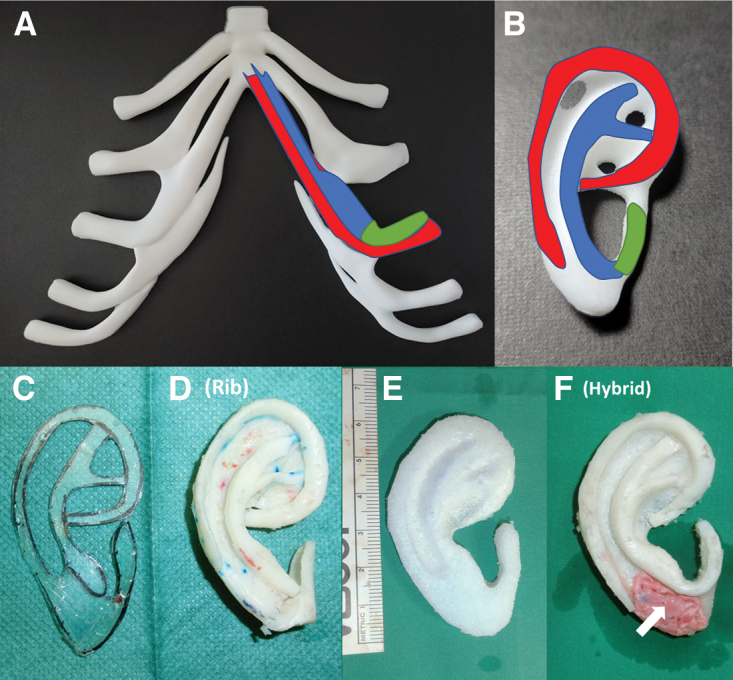
Surgical technique for reconstruction of the auricular framework by rib cartilage alone or combination rib cartilage and porous polyethylene. **(A)** Rib cartilage was harvested from the 7^th^ rib on either the ipsilateral or contralateral side of the chest. **(B)** The helix (red), antihelix (blue) and antitragus (green) were formed from either side of the lateral border of the 7^th^ rib cartilage. **(C)** The template of the ear is copied from the opposite ear. **(D)** An auricular framework is formed from autologous rib cartilage (ACC). **(E)** The base of the auricular framework was constructed from either a porous high-density polyethylene “Siegert” auricular prosthesis (Stryker, Kalamazoo, MI) with the helix and antihelix shaved out, or carved from a polyethylene implant block [63 mm (Length) × 30 mm (Width) × 6 mm (Thickness)] (Stryker, Kalamazoo, MI) using a surgical burr and scalpel. The scaphoid fossa, triangular fossa, concha symba and concha cavum are formed with a cutting burr. **(F)** A hybrid auricular framework is formed by binding rib cartilage to the base of the polyethylene implant with stainless-steel wire to form a helix, antihelix and tragus. Perichondrium (white arrows) is placed to cover the polyethylene implant in the region of the earlobe.

**Fig. 2.**
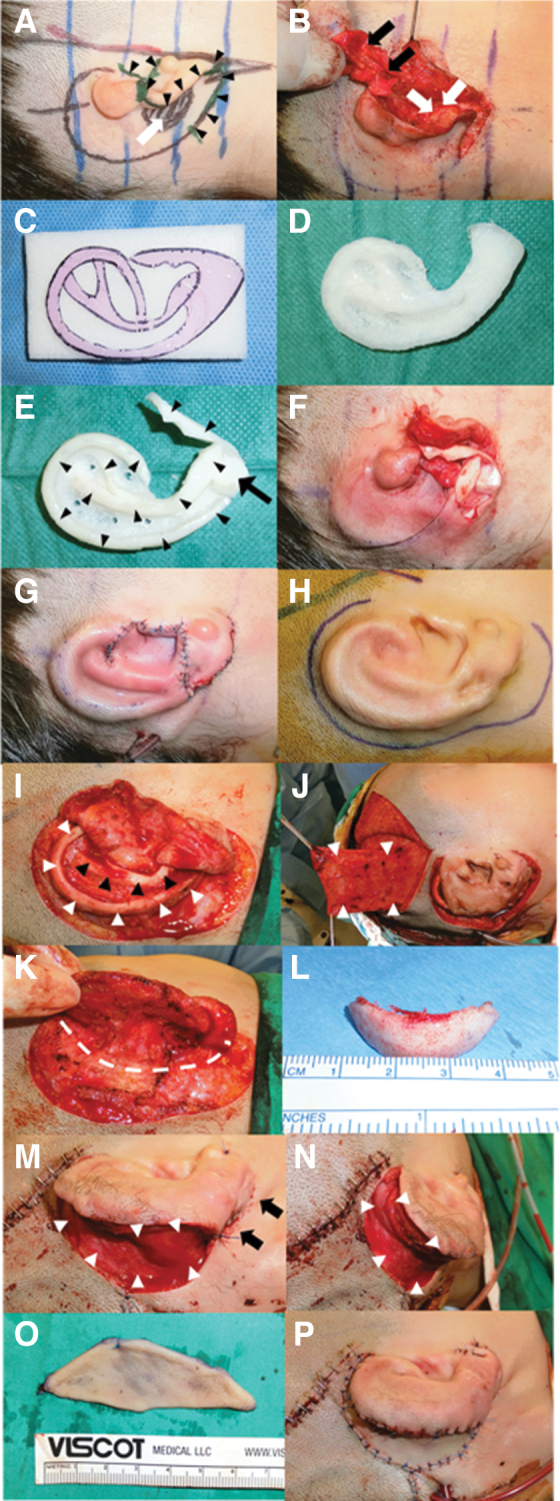
Intraoperative photographs showing microtia reconstruction by a modified Nagata's technique for stage 1 and stage 2 operations using rib cartilage and a porous polyethylene. **(A)** An ‘S’ incision (small black arrows) is made in the preauricular, auricular and postauricular region to prepare for the elevation of the skin flap and to allow transposition of the earlobe. A skin pedicle attached to the mastoid region is preserved (black shaded area with white arrow). The position and orientation of the auricular framework is planned according to the opposite ear. **(B)** The skin flap is elevated with a skin pedicle remaining attached to the mastoid fascia (white arrows) which allows the earlobe to be transposed. The remnant of the ear cartilage is removed (black arrows). **(C)** A template of the opposite ear is prepared using a plastic film to facilitate carving of the porous high-density polyethylene implant block. **(D)** The base of the auricular framework is carved from a polyethylene block to form a scaphoid fossa, triangular fossa, concha symba and concha cavum. **(E)** Addition of the helix, antihelix, antitragus and tragus by autologous rib cartilage (small black arrows) to the polyethylene implant to form a hybrid graft. Rib cartilage is also added to cover the earlobe region of the framework (large black arrows). **(F)** Placement of the hybrid auricular framework deep to the skin pocket with the pedicle of the skin flap located in the concha cavum. **(G)** The shape of the auricle after placement of the hybrid auricular framework and transposition of the earlobe. Redundant skin is excised. **(H)** Appearance of the auricle after stage 1 reconstruction while undergoing stage 2 reconstruction to create auricular projection. **(I)** The auricular skin is peeled off from the hybrid auricular framework to allow excision of redundant fibrofatty tissue in the scaphoid fossa and triangular fossa. An intact helix (white arrows) and antihelix (black arrows) by autologous rib cartilage and good bio-integration of the soft tissue to the polyethylene base are seen. **(J)** A temporoparietal fascial flap is harvested from the temporal region by an external skin incision. **(K)** Elevation of the hybrid auricular framework and the skin from the mastoid to create a postaural sulcus (white dotted line). **(L)** A wedge of polyethylene implant is carved according to the requirement for projection based on the opposite ear. **(M)** The polyethylene wedge is placed in the postaural sulcus to check the auricular projection which is then covered by vascularized temporoparietal fascia (white arrows). The cervical skin is advanced superiorly to cover the postaural defect posterior to the earlobe (black arrows). **(N)** The projection of the hybrid auricular framework is achieved after placement of the polyethylene wedge covered by vascularized temporoparietal fascia (white arrows). **(O)** A full thickness skin graft is harvested from the lower abdomen and the wound closed primarily. **(P)** The immediate postoperative appearance of the auricle after Nagata s stage 2 reconstructive surgery with a closed suction drain to prevent a hematoma and to maintain negative pressure for better skin binding to the auricular framework.

Figure 1 summarizes our technique for construction of the rib and hybrid auricular frameworks. For patients with bilateral microtia, the seventh rib cartilage was used to form the helices and tragus, and the eighth rib to build the antihelices and antitragus on both hybrid auricular frameworks. The hybrid auricular framework was soaked in gentamicin solution (80 mg in 20 mL of normal saline) before implantation. Figure 2 summarizes the surgical steps of our modified Nagata's stage 1 microtia reconstruction using a hybrid auricular framework, and our modified Nagata's stage 2 microtia reconstruction using an implant wedge covered with vascularized temporoparietal fascia and a full thickness skin graft.

### Microtia aesthetic outcome evaluation

Preoperative and 12-month postoperative photographs were rated by 4 investigators, 2 senior otolaryngologists and 2 surgical residents, blinded to the surgical technique. Each investigator rated the aesthetic outcome of the auricle twice, based on a guideline (Supplementary Table A), with at least a 1-day interval that allowed evaluation of the intrarater and inter-rater reliability of the scores. The anterolateral oblique view of the auricle before and after microtia reconstruction was evaluated for size, shape, skin color, scar, projection of the auricle, and conchal depth.

The posterolateral oblique view of the auricle after microtia reconstruction was used to evaluate projection, depth of the postaural sulcus, and the postauricular scar. The postoperative aesthetic outcome of the auricle was rated for skin color, shape, concha, projection, postaural sulcus, three-dimensional appearance, wound scarring, auricular hairs, hair loss, and overall improvement. Each item was rated on a 4-point scale as excellent (3), good (2), fair (1), or poor (0).

### Outcomes and statistical analysis

The interpretation of the inter- and intraclass correlation coefficient (ICC) for the four investigators of the microtia aesthetic score was based on the publication by Koo and Li.^19^ The Mann–Whitney *U* test was used to compare continuous values and Fisher's exact test categorical data for statistical differences between the rib and the hybrid groups. The Mann–Whitney *U* test was used to compare the total microtia aesthetic outcome scores and the scores for their individual items in the rib and hybrid groups. A *p*-value of <0.05 was considered significant.

## Results

**Fig. 3.**
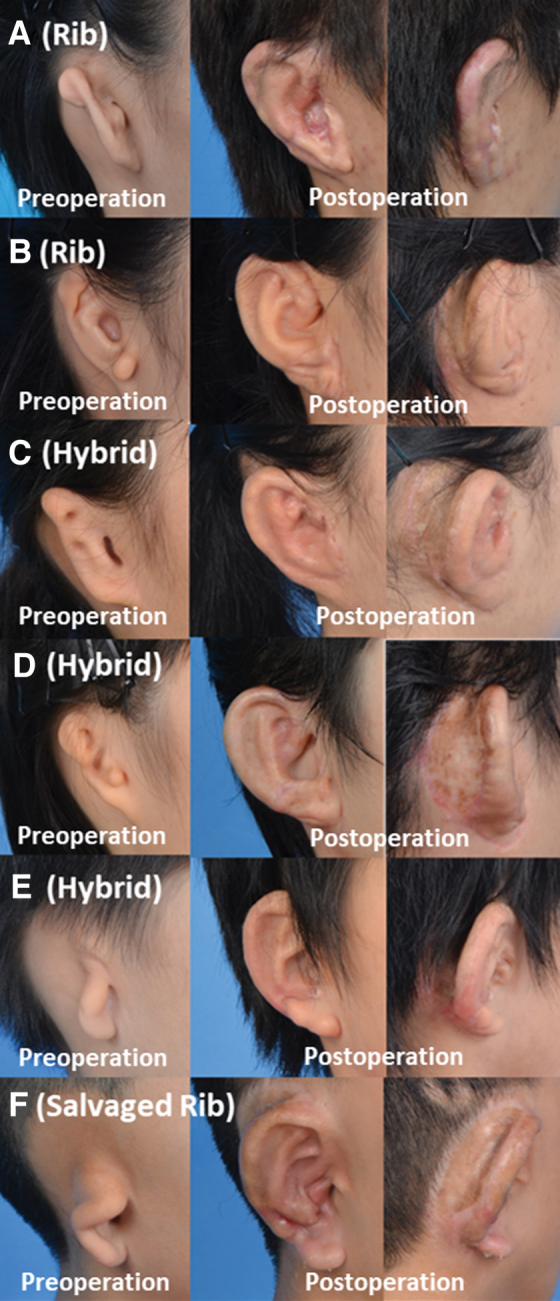
Appearance of the auricle before and after microtia reconstruction using the rib cartilage and hybrid auricular framework made with a rib cartilage and porous polyethylene. **(A)** (Left): Lobular type of microtia. (Middle): Reconstruction by a rib auricular framework. (Right): Postaural sulcus. **(B)** (Left): Small conchal type of microtia. (Middle): Reconstruction by a rib auricular framework. (Right): Postaural sulcus. **(C)** (Left): Small conchal type of microtia. (Middle): Reconstruction by a hybrid auricular framework. (Right): Postaural sulcus. **(D)** (Left): Lobular type of microtia. (Middle): Reconstruction by a hybrid auricular framework. (Right): Postaural sulcus. **(E)** (Left): Lobular type of microtia. (Middle): Reconstruction by a hybrid auricular framework. (Right): Postaural sulcus. **(F)** (Left): Large conchal type of microtia. (Middle): Revision reconstruction by a rib auricular framework as salvage surgery after explantation of a hybrid auricular framework caused by persistent infection. (Right): Postaural sulcus.

Forty-three patients with 47 ears underwent microtia reconstruction using the classical or modified Nagata's technique between 2012 and 2019. Three patients with unilateral microtia were excluded due to incomplete follow-up or photographic documentation. Four patients had bilateral microtia reconstruction using a hybrid auricular framework during a single operating session. Forty patients and 44 ears were eligible for review and analysis. Eleven ears were reconstructed with a rib auricular framework **(**Fig. 3A, B**)** and 33 with a hybrid auricular framework **(**Fig. 3C, D**)**.

There were 26 male and 14 females, with a mean age of 14.2 years (8–29 years). The mean age of the patients in the hybrid group was 3.1 years younger than that in the rib group, as patients with a minimum age of 8 years were eligible for microtia reconstruction if a hybrid auricular framework was used, whereas a minimum age of 12 years was required for the rib group. The operating times for Nagata's stage 1 and stage 2 reconstructions in the hybrid group were 2.6 and 1.4 h shorter than those for the rib group, respectively.

The mean postoperative follow-up was 76.3 months (20–104 months) for the rib group and 43.1 months (14.8–95 months) for the hybrid group. Table 1 summarizes the patient demographic data, microtia condition, operative time, postoperative time, and follow-up interval.

**Table 1.** Patient characteristics and preoperative, intraoperative, and postoperative conditions of the autologous rib cartilage and hybrid framework group

	Autologous rib cartilage	Hybrid	*p*
Number of patients	11	29	—
No. of reconstructed ears	11	33	—
Age (year)	16.5 (12–24)	13.4 (8–29)	0.025
Gender: male versus female	8:3	20:13	0.72
Right versus left ear	8:3	21:12	0.72
Microtia: lobular versus concha	4:7	20:13	0.19
Ear canal atresia	8 (72.7%)	23 (69.7%)	1
Implantable hearing device	3 (27.3%)	8 (24.2%)	1
Postoperative follow-up time (months)	76.3 (20–104)	43.1 (14.8–95)	0.005
Stage 1 operating time (hours)	9.5 (6.5–13.1)	6.9 (3.5–9.6)	<0.001
Stage 2 operating time (hours)	7.8 (5.2–10.9)	6.4 (4.8–8.4)	0.016
Stages 1 and 2 time interval (months)	10.2 (6.4–16.3)	9.2 (6.2–15.7)	0.29
Length of auricle (mm)	62.7 (55–65)	61.6 (55–69)	0.19
Total aesthetic outcome score (overall)	26.4	27.9	0.40
Total aesthetic outcome score (bilateral microtia)	—	29.3	—

*p*-Value is significant if <0.05.

Table 2 summarizes the early and late postoperative complications in the rib and hybrid auricular framework groups. There was no statistically significant difference between the two groups. Twelve ears had skin flap necrosis after stage 1 reconstruction, six of them (50%) required local skin flaps to cover the auricular framework. Seven patients (21.2%) in the hybrid group had secondary infections, stainless-steel wire extrusion, framework exposure, auricle trauma, auricular skin, and/or branchial fistula infection.

**Table 2.** Postoperative complications in autologous rib cartilage and hybrid auricular framework

	Autologous rib cartilage	Hybrid	*p*
Pneumothorax	0	0	1
Skin necrosis (poststage 1)	3 (27.2%)	9 (27.2%)	1
Concha	3	5	0.39
Helix	1	1	0.44
Earlobe	0	4	0.56
Local skin flap	2 (18.2%)	4 (12.1%)	0.63
Infection	0	7 (21.2%)	0.17
Postoperation	0	1	
Wire extrusion	0	1	
Framework exposure	0	2	
Ear injury	0	1	
Others: Skin, fistula	0	2	
Wire extrusion	1 (9.1%)	6 (18.2%)	0.66
Fascia reinforcement	0	5	0.31
Framework exposure	1 (9.1%)	5 (15.2%)	0.66
Poststage 2 interval (month)	33.9	28.5 (10.6–37.8)	0.38
Helix	1	1	
Antihelix	0	2	
Antitragus	0	1	
Triangular fossa	0	1	
Treatment	0	1 LF, 4 FR	0.66
Ear framework fracture	0	0	
Hypertrophic scar (steroid injection)	4 (36.4%)	14 (42.4%)	1
Keloid scar in ear	1 (9.1%)	0	0.25
Laser hair removal	8 (72.7%)	20 (60.6%)	0.72
Framework explantation	0	5 (15.2%)	0.31
Infection	0	3 (9.1%)	
Exposure	0	1 (3%)	
Branchial fistula infection	0	1 (3%)	

*p*-Value is significant if <0.05.

FR, fascia reinforcement; LF, local flap.

Stainless-steel wire extrusion occurred in seven ears (15.5%), one in the rib group and six in the hybrid group. In the hybrid group, skin closure after removal of extruded wires was reinforced with postaural temporoparietal fascia. Auricular framework exposure occurred in six ears (13.6%): one (9.1%) in the rib group due to auricle trauma and five (15.2%) in the hybrid group due to auricle trauma, resorption of rib cartilage, neglected stainless-steel wire extrusion, or partial loss of temporoparietal fascia. No postaural implant wedges became exposed or were extruded during follow-up.

One ear in the hybrid group suffered a sports-related injury to the auricular skin exposing the framework that required a local skin flap for cover. Another four exposed hybrid frameworks were treated by skin wound closure reinforced with postaural temporoparietal fascia (Supplementary Fig. A). One auricular framework in the rib group exposed due to trauma healed spontaneously. Five hybrid auricular frameworks in three patients required explantation of which four ears were from two bilateral microtia patients. They were 11, 15, and 16 years old and explanted on average at 18.6 months (2–28 months) after stage 2 reconstruction.

One was due to postoperative infection, one secondary to infection caused by an unattended framework exposure, one due to branchial fistula infection, and two framework exposures in a bilateral microtia patient with partial loss of temporoparietal fascia on one side and resorption of rib cartilage with extrusion of stainless-steel wires on the other, and who declined revision surgery with rib cartilage. Two ears were successfully revised using a rib auricular framework and a rib cartilaginous wedge placed into the pre-existing soft tissue and skin envelope after explantation **(**Fig. 3E**)**. One ear was salvaged by a prosthetic ear and two ears had no further treatment after auricular explantation.

The total score for the microtia aesthetic outcome evaluations for the 44 reconstructed auricles by the four investigators demonstrated a moderate-to-excellent intrarater reliability (ICC: 0.56–0.92) and a good inter-rater reliability (ICC: 0.88, 95% confidence interval: 0.74 – 0.94). The mean total microtia aesthetic outcome score for the hybrid group (27.9) was slightly better than for the rib group (26.4), but with no statistical difference. The mean total microtia aesthetic outcome score for the four bilateral cases using hybrid auricular frameworks (29.3) was better than the rib (26.4) and hybrid groups (27.9), respectively **(**Table 1**)**.

There was also no statistical difference between most of the individual items of the microtia aesthetic outcome score for the rib and hybrid groups (Supplementary Table B). Hypertrophic scar was the only parameter showing a statistical difference in the microtia aesthetic score between the rib group (2.09) and hybrid group (2.45), although both scores were in the “good” category.

## Discussion

Microtia reconstruction is typically performed in the first 10 years of life with either rib cartilage or an implant like porous polyethylene, with the later avoiding a rib graft donor site and allowing for an earlier surgery. Our study evaluated the outcome of a hybrid framework of an implant with rib cartilage using Nagata's technique for microtia reconstruction. Forty-four ears of 40 patients underwent surgery, 11 ears received a rib auricular framework and 33 ears a hybrid auricular framework. Stainless-steel wires extruded in seven ears (15.9%) and five (15.2%) hybrid frameworks were removed due to infection or extrusion. Aesthetic outcomes were similar for both groups.

The small number of subjects in our series may account for apparently high complication rates. Although the incidence of framework exposure and explanation in the hybrid group was 15.2%, these preliminary results are within the published range of 3.7–30% for implant exposure and 0–17.6% for implant explantation despite protection by vascularized temporoparietal fascia.^9,20,21^ However, we believe these rates can be lowered by addressing some of the responsible factors before and after surgery.

In our series, unattended stainless-steel wire extrusion is the main cause of framework exposure and infection. This is due to minor resorption of rib cartilage, which protects the implant, resulting in protrusion of the stainless-steel wires and irritation of the overlying skin. This can be reduced by increasing the thickness of the rib cartilage grafts for the helix, antihelix, and antitragus, and by embedding the stainless-steel wires deeply into the cartilage when binding them to the implant. The high incidence of infection in the hybrid group (21%) was mostly due to stainless-steel wire extrusion and framework exposure. However, an implant is generally quite resistant to infection, and most infections were treated by wound exploration and removal of extruded wires, cleansing with betadine solution, and covering of any exposed framework with postaural temporoparietal fascia.

In selecting a surgical approach, it is important to know which patients are unable to attend regular postoperative follow-ups due to geographical reasons. Such patients may not be suitable for a hybrid approach unless a mutually acceptable postoperative follow-up plan can be agreed on. We observed more complications including explantation in subjects who did not live locally and who missed their regular follow-up. We suggest patients are followed up regularly for at least 2 years after surgery, which is the crucial period when most stainless-steel wire or framework exposures occur, and that are easier to manage when they are identified early.

The framework was exposed in two subjects after injury while playing a sport. Although we do not discourage sports after microtia reconstruction, subjects who do play sport, especially where physical contact is possible, should be warned of the potential sequelae of ear trauma that may be difficult to salvage and thus require immediate attention.

One advantage of our hybrid approach is that it allows reconstruction of microtia using the classical Nagata's two-stage technique without major changes to the surgical method when the traditional auricular framework is constructed with rib. Moreover, our hybrid technique requires less rib cartilage to be harvested, and harvested only once at the first operation. The mean operating time for the hybrid group was 2 h shorter than for the rib group in stage 1 reconstruction. For bilateral microtia reconstructions, the seventh and eighth rib cartilages were sufficient for constructing two hybrid auricular frameworks for bilateral implantation during the same operating session.

The operating time was shorter for the hybrid group as less rib cartilage is needed to be harvested. Lastly, revision reconstruction by a rib auricular framework as salvage is feasible using the pre-existing skin envelope after explantation of the hybrid framework provided all infection is controlled.

By using an implant, the minimum age of our patients for reconstruction could be lowered from 12 to 8 years when the sizes of their auricles have attained 85% of their projected adult size based on our previous study of >250 subjects.^22^ Our results showed that both the rib group and hybrid groups had no significant difference in the total microtia aesthetic outcome assessment scores. The hybrid technique provides another option for surgeons to consider in their armamentarium for microtia reconstruction that allows surgery to be performed with predictable and reproducible outcomes in younger patients or in bilateral cases, requiring less rib cartilage to be harvested at one surgery only. However, rib cartilage is still considered as a standard reconstruction for older patients.

## Conclusion

A hybrid auricular framework, formed from a combination of an implant with rib cartilage, is a feasible option for reconstructing congenital microtia, and is, therefore, a modification of Nagata's surgical technique that traditionally uses rib cartilage only. This modification reduces the operating time and the amount of rib cartilage that needs to be harvested, and limits harvesting to one operation only. The aesthetic outcomes are similar for both the traditional rib and hybrid reconstructions. However, caution should be used when choosing the hybrid versus the rib-only approach as the explanation rate was higher in the hybrid group in this study.

## Supplementary Material

Supplemental data

Supplemental data

Supplemental data
