# Supplementary material for: A Hybrid Auricular Framework of Autologous Rib Cartilage and a Porous Polyethylene Implant for Reconstruction of Congenital Microtia: A Modification of Nagata's Technique
Source: Facial Plast Surg Aesthet Med. 2024 Jan 8;26(1):15–22. doi: 10.1089/fpsam.2022.0152 (PMC10794839; doi:10.1089/fpsam.2022.0152)
Supplement: Supplemental data [file Suppl_TableA.docx]

**Supplementary Table A:** Scoring system for microtia surgery outcomes

| 1. **Auricular skin color - How good does the auricular skin color match the color of the facial skin**    - 3 – Excellent: Perfect match    - 2 – Good: Mildly deeper / lighter than facial skin    - 1 – Fair: Obvious deeper or lighter than facial skin    - 0 – Poor: Very big contrast with facial skin with patchy hyper or hypopigmentation |
| --- |
| 1. **Auricular shape - How smooth is the outline of the auricle**    - 3 – Excellent: Very smooth, upper, mid, and lower ear are in proportion    - 2 – Good: Mild breaking of contour in the border of ear with depression or bumping    - 1 – Fair: Obviously missing a part of the ear in upper, middle or lower    - 0 – Poor: A deformed shape far away from a normal ear |
| 1. **Auricular features - The presence of helix, antihelix with superior and inferior crus, tragus, antitragus, earlobe**    - 3 – Excellent: All features are present    - 2 – Good: 1 or 2 structures is/are not obvious    - 1 – Fair: Missing 2 or more structures but is still barely perceived as an ear    - 0 – Poor: Featureless auricle |
| 1. **Concha**    - 3 – Excellent: Deep and looks as if an ear canal opening is present    - 2 – Good: Obviously with a depression but still not deep enough to be perceived as having an ear canal opening    - 1 – Fair: A mild depression only    - 0 – Poor: absent and no such feature is perceived |
| 1. **Auricular projection - How good is the projection of the ear from the scalp**    - 3 – Excellent: Auricle is obviously hanging out from the mastoid region    - 2 – Good: Auricle has an acceptable elevation from the mastoid    - 1 – Fair: Auricle is barely elevated from the mastoid    - 0 – Auricle looks as if it is still buried under the scalp |
| 1. **Postaural sulcus - How good is the sulcus formed between the auricle and the mastoid**    - 3 – Excellent: A deep postaural sulcus from upper part of the auricle down to the earlobe    - 2 – Good: A deep postaural sulcus from upper to middle part of the auricle    - 1 – Fair: A sulcus is barely perceived in any part of the ear    - 0 – Poor: No postaural sulcus can be seen at all |
| 1. **Overall 3-dimensional appearance of the auricle - An overall measure of the prominence of the helix, antihelix and conchal bowl formed by the depth of the scaphoid fossa, triangular fossa, concha symba and concha cavum**    - 3 – Excellent: Very good 3D appearance of auricle due to an adequate depth of those fossae of the auricle    - 2 – Good: Reasonable depression those fossae to give some 3D perception of the auricle    - 1 – Fair: Barely seen or doubtful presence of depression in those fossae of the auricle    - 0 – Poor: No depression seen in those sunken area of the auricle |
| 1. **Hypertrophic scar/keloid - Abnormal wound healing with scarring seen in the wound of the auricle**    - 3 – Excellent: None is seen    - 2 – Good: Mild and not affect the aesthetic of the auricle    - 1 – Fair: Obvious and causing mild degree of    - 0 – Poor: Severe causing gross deformity of the auricle |
| 1. **Auricular hairs - Any residual hair in the lateral auricular skin**    - 3 – Excellent: none    - 2 – Good: Scanty thin hair only in the helix    - 1 – Fair: Thin hair in helix and antihelix    - 0 – Poor: Obvious patchy thick hair all over the auricle |
| 1. **Hair loss - Hair loss in the temporal scalp after harvesting of the temporoparietal fascia**    - 3 – Excellent: No perceivable hair loss    - 2 – Good: Mild thinning of hair in some area of the temporal scalp is seen    - 1 – Fair: Obvious patchy hair loss is seen    - 0 – Poor: Obvious patchy hair loss and thinning of hair in many areas of the scalp in temporal region |
| 1. **Overall Rating - How do you rate the overall result and improvement when compared to the preoperative appearance of the ear**    - 3 – Excellent: Outstanding result with impressive improvement in the auricle when compared to preoperative appearance    - 2 – Good: Good result with a decent improvement in the auricle, not perfect but much better than preoperatively.    - 1 – Fair: Acceptable shape of the ear without many features, not consider as pretty at all. Just pass but no need for revision    - 0 – Poor: Shape far away from appearance of a ear, a failed reconstruction, strong desire of “redo” |
