# Supplementary material for: A Hybrid Auricular Framework of Autologous Rib Cartilage and a Porous Polyethylene Implant for Reconstruction of Congenital Microtia: A Modification of Nagata's Technique
Source: Facial Plast Surg Aesthet Med. 2024 Jan 8;26(1):15–22. doi: 10.1089/fpsam.2022.0152 (PMC10794839; doi:10.1089/fpsam.2022.0152)
Supplement: Supplemental data [file Suppl_FigureA.docx]

**Supplementary Figure A S1: (A)** Framework exposure of a hybrid auricular framework due to scar contracture in the triangular fossa of a male patient 15 months after stage 2 reconstruction. Preauricular skin incision marked by a dotted line and the advantageous placement of the fascia. **(B)** Skin incision and elevation of the skin flap to expose the triangular fossa and inferior crus of antihelix (long white arrow) with moderate resorption of rib cartilage previously placed on the porous high-density polyethylene implant. Rib cartilage was intact in the helix (white arrows) over the polyethylene auricular base (black arrows). Wound closure reinforced with postaural temporoparietal fascia for a hybrid framework exposure in the triangular fossa region. **(C)** Harvesting of the postaural temporoparietal fascia (white arrow) transposed from the temporal region in a previous stage 2 reconstruction. **(D)** Thick temporoparietal fascia ready to facilitate wound closure. **(E)** Placement of the temporoparietal fascia in the triangular fossa and inferior crus of the antihelix to reinforce skin wound closure. No sutures or glue were needed to secure the position of the fascia. **(F)** Closure of the preauricular skin wound and the dehiscent skin wound over the triangular fossa by non-absorbable sutures. No pressure dressing was needed after meticulous hemostasis. **(G)** Three days after surgery showing no collection under the skin. **(H)** Three weeks after skin wound closure and reinforcement. The skin wound was completely healed, and the closure reinforced by underlying temporoparietal fascia.**
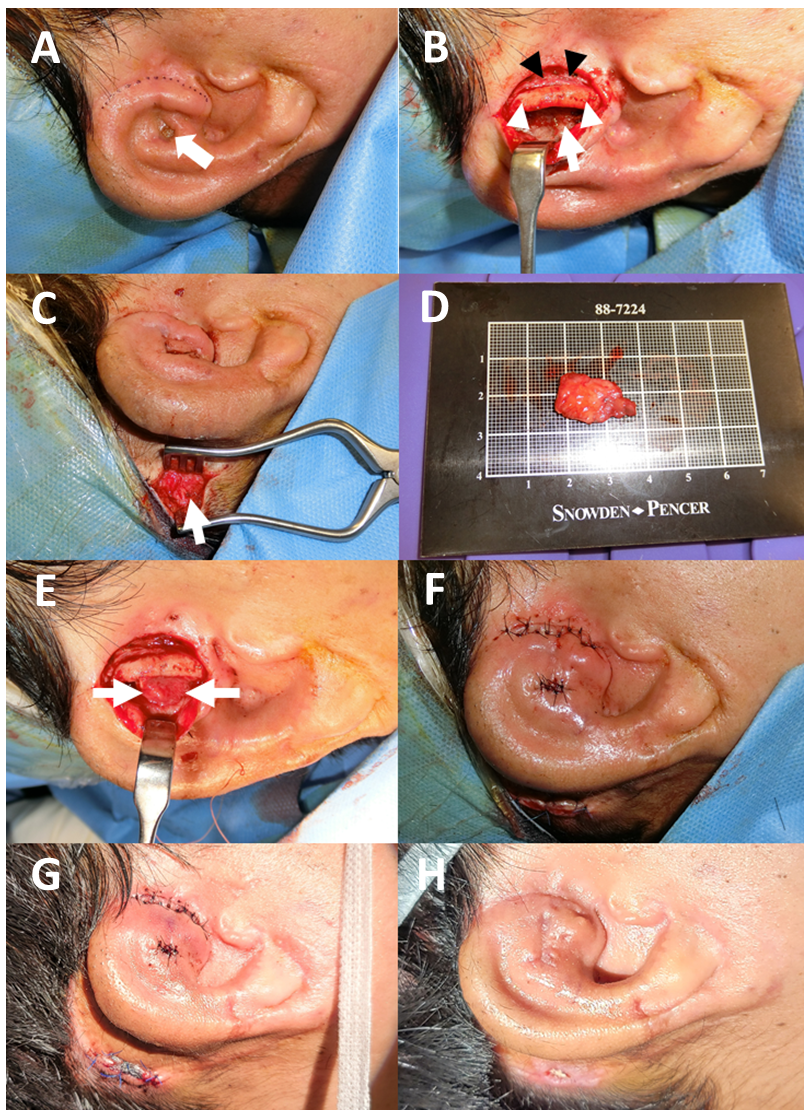
**
