# Supplementary material for: A Hybrid Auricular Framework of Autologous Rib Cartilage and a Porous Polyethylene Implant for Reconstruction of Congenital Microtia: A Modification of Nagata's Technique
Source: Facial Plast Surg Aesthet Med. 2024 Jan 8;26(1):15–22. doi: 10.1089/fpsam.2022.0152 (PMC10794839; doi:10.1089/fpsam.2022.0152)
Supplement: Supplemental data [file Suppl_TableB.docx]

**Supplementary table B:** Mean score of parameters for the evaluation of microtia reconstruction using autologous rib cartilage and hybrid auricular frameworks

|  | **Group** | **N** | **Mean Score** | **Standard Deviation** | **p value** |
| --- | --- | --- | --- | --- | --- |
| **Shape** | Rib cartilage | 11 | 2.36 | 0.48 | 0.11 |
|  | Hybrid | 33 | 2.62 | 0.32 |  |
| **Features** | Rib cartilage | 11 | 2.17 | 0.56 | 0.13 |
|  | Hybrid | 33 | 2.44 | 0.42 |  |
| **Concha** | Rib cartilage | 11 | 2.22 | 0.61 | 0.83 |
|  | Hybrid | 33 | 2.29 | 0.48 |  |
| **Projection** | Rib cartilage | 11 | 2.67 | 0.39 | 0.77 |
|  | Hybrid | 33 | 2.66 | 0.38 |  |
| **Postaural sulcus** | Rib cartilage | 11 | 2.20 | 0.59 | 0.24 |
|  | Hybrid | 33 | 2.45 | 0.40 |  |
| **3-Dimension** | Rib cartilage | 11 | 2.45 | 0.59 | 0.80 |
|  | Hybrid | 33 | 2.51 | 0.39 |  |
| **Skin color** | Rib cartilage | 11 | 2.66 | 0.34 | 0.29 |
|  | Hybrid | 33 | 2.78 | 0.23 |  |
| **Hypertrophic scar** | Rib cartilage | 11 | 2.09 | 0.55 | 0.04 |
|  | Hybrid | 33 | 2.45 | 0.38 |  |
| **Auricular hair** | Rib cartilage | 11 | 2.88 | 0.17 | 0.26 |
|  | Hybrid | 33 | 2.91 | 0.20 |  |
| **Scalp hair loss** | Rib cartilage | 11 | 2.68 | 0.58 | 0.60 |
|  | Hybrid | 33 | 2.67 | 0.45 |  |
| **Overall improvement** | Rib cartilage | 11 | 2.33 | 0.57 | 0.52 |
|  | Hybrid | 33 | 2.49 | 0.36 |  |
| **Total Score** | Rib cartilage | 11 | 26.39 | 4.22 | 0.40 |
|  | Hybrid | 33 | 27.91 | 2.37 |  |
